# Sarcoïdose pulmonaire en lâcher de ballon: à propos d’un cas

**DOI:** 10.11604/pamj.2016.24.295.7664

**Published:** 2016-08-03

**Authors:** Haykel Abdelhedi, Naziha Khammassi, Amira Mhenni, Youssef Kort, Ouahida Cherif

**Affiliations:** 1 Service de Médecine Interne, Hôpital Razi, la Manouba 2010, Faculté de Médecine de Tunis, Tunis Tunisie

**Keywords:** Sarcoïdose, lâché de ballon, atteinte pulmonaire, Sarcoidosis, multiple scattered pulmonary nodules, pulmonary involvement

## Abstract

La sarcoïdose est une granulomatose multisystémique pouvant toucher tous les organes, l'atteinte thoracique est la plus fréquente et la plus évocatrice. Elle peut prendre des aspects atypiques, dont l'aspect en lâcher de ballon pulmonaire faisant craindre une pathologie maligne. Patiente âgée de 56 ans, était explorée pour une asthénie associée à des paresthésies des membres inférieurs évoluant depuis 6 mois. Le scanner thoracique montrait un aspect en lâcher de ballon. L'examen histologique de la biopsie bronchique avait permis d'éliminer une origine néoplasique et de confirmer le diagnostic de sarcoïdose. Du fait des explorations fonctionnelles respiratoires normales et de l'absence d'atteinte viscérale extra-pulmonaire une simple surveillance a été proposée. La sarcoïdose peut exceptionnellement donner des images radiologiques en lâcher de ballon. La preuve histologique est nécessaire pour éliminer d'autres étiologies notamment tumorales. La prescription d'une corticothérapie ne paraît indiquée qu'en présence d'un retentissement fonctionnel respiratoire significatif et/ou d'une atteinte viscérale associée.

## Abstract (en)

Sarcoidosis is a multisystem granulomatous disease that can affect all organs, thoracic involvement often predominates representing its most evocative manifestation. Atypical manifestations, such as the presence of multiple scattered pulmonary nodules may indicate malignancy. Our study reports the case of a 56-year-old patient investigated for asthenia associated with paresthesia of the lower limbs evolving since 6 months. Chest CT scan showed multiple scattered pulmonary nodules. Histological examination of specimens obtained by bronchial biopsy allowed to exclude neoplastic origin and confirmed the diagnosis of sarcoidosis. On the basis of normal pulmonary function tests and the absence of extrapulmonary visceral involvement, simple patient monitoring was recommended. Radiologically sarcoidosis can exceptionally present as multiple scattered pulmonary nodules. Histological evidence is necessary to exclude other etiologies including tumors. Corticosteroids are indicated only in the presence of severe respiratory functional impairment and/or associated visceral involvement.

## Introduction

La sarcoïdose est une granulomatose systémique de cause inconnue, pouvant toucher tous les organes, caractérisée par la formation de granulomes immunitaires [1]. L'atteinte thoracique est la plus fréquente [2]. Le diagnostic est évoqué devant un tableau clinico-radiologique compatible mais la preuve reste histologique avec la présence de granulomes épithélioïdes et giganto-cellulaires sans nécrose caséeuse permettant d'écarter d'autres diagnostics [1]. Elle s'accompagne d'une atteinte pulmonaire et/ou ganglionnaire thoracique dans 90% des cas [3]. Des anomalies sont présentes à la radiographie thoracique dans 85 à 95% des cas, le plus souvent typiques ou fortement évocatrices. Elle peut prendre des aspects atypiques, dont l'aspect en lâcher de ballon pulmonaire faisant craindre une pathologie maligne. Cette forme pseudotumorale, pourtant rare constitue un piège à prendre en considération dans l'approche diagnostique [1, 4]. Nous rapportons le cas d'une patiente avec une présentation de ce type de sarcoïdose pulmonaire.

## Patient et observation

**Figure 1:**
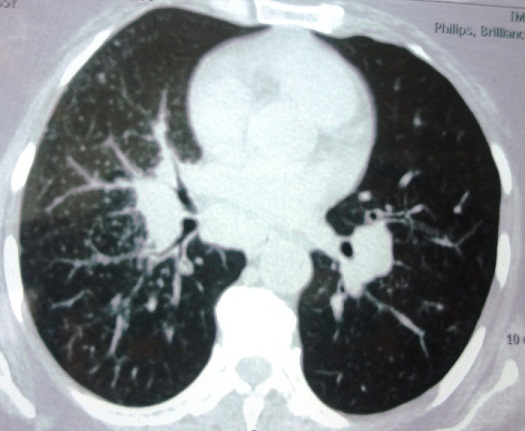
Scanner thoracique: adénopathies médiastinales asymétriques, sous carinaires et hilaires, associées à de multiples nodules pulmonaires apicaux et ventraux et à des micronodules centrolobulaires diffuse

Il s'agit d'une femme de 56 ans, non tabagique, allergique à la pénicilline, aux antécédents de coliques néphrétiques et de kyste de l'ovaire opéré, qui a été hospitalisée pour une asthénie associée à des paresthésies des membres inférieurs évoluant depuis 6 mois sans altération de l'état général. L'examen physique était sans particularités. La biologie a objectivé une anémie hypochrome microcytaire (Hb à 10,6g/dl) sans syndrome inflammatoire ni perturbation du bilan hépatique ou de l'ionogramme sanguin. Le bilan phosphocalcique a objectivé une calcémie normale à 2,38 mmol/l (N: 2,15-2,57 mmol/l) associée à une augmentation de la calciurie à 6.25 mmol/24h (N: 1,2-3,7 mmol/24h) et de l'enzyme de conversion de l'angiotensine à 98 UECA (N: 12-68 UECA). La radiographie thoracique a objectivé deux gros hiles pulmonaires. Le scanner thoraco-abdomino-pelvien a révélé des adénopathies médiastinales asymétriques, sous carinaires et hilaires bilatérales profondes et compressives, associées à de multiples nodules pulmonaires apicaux et ventraux du lobe supérieur droit, sous pleuraux du culmen, du segment interne du lobe moyen, sous pleuraux postéro basal droit et du segment inférieur de la lingula, de diamètre variant entre 5 et 10 mm. Ces nodules étaient associés à des micronodules centrolobulaires diffus prédominant au niveau du lobe moyen réalisant par endroit un aspect d'arbre en bourgeons (Figure 1). La fibroscopie bronchique a objectivé un aspect granulomateux diffus. La biopsie trans-bronchique a été pratiquée et l'examen histologique a montré une inflammation épithélioïde et giganto-cellulaire sans nécrose caséeuse. La recherche de bacille de Koch dans les crachats était négative.

Le diagnostic de sarcoïdose a été retenu devant l'hypercalciurie, l'élévation de l'enzyme de conversion, l'aspect radiologique et les données de l'histologie bronchique. Le lavage broncho-alvéolaire était normocellulaire (53*10^3^/ml) avec une lymphocytose modérée à CD4 (CD4/CD8 à 4,5). L'exploration fonctionnelle respiratoire était normale (VEMS = 78%, CVF = 82%). Une atteinte hépatique ou splénique a été écartée devant l'absence d'anomalies biologiques évocatrices et de viscéromégalie à l'échographie abdominale. L'électrocardiogramme pratiqué à la recherche d'une atteinte cardiaque était normal ainsi que l'électromyogramme. Il n'y avait pas de lésions cutanées évocatrices, ni d'atteinte oculaire à l'examen ophtalmologique demandé systématiquement. Du fait des explorations fonctionnelles respiratoires normales et de l'absence d'atteinte viscérale extra-pulmonaire une simple surveillance a été proposée.

## Discussion

Au cours de la sarcoïdose, l'atteinte médiastino-pulmonaire constitue la manifestation la plus fréquente et quasiconstante. Celle-ci revêt des phénotypes radio-cliniques très divers mais assez souvent évocateurs du diagnostic [2]. Il existe, toutefois, certaines présentations déroutantes pouvant évoquer d'autres diagnostics différentiels. On en cite les atteintes pulmonaires dites pseudotumorales qui, en dépit de leur rareté, méritent d'être prises en considération. La fréquence de ces formes varie selon les séries entre 6 et 34% [2, 5]. Depuis la première publication par Mac Cord & Hyman en 1952 [5], on en dénombre moins de 50 cas publiés [6] jusqu'à ce jour. La découverte radiologique de ces nodules multiples disséminés fait évoquer les lésions tumorales (métastases, lymphomes) en première intention et les infections (tuberculose, hydatidose…) en deuxième intention [1, 7]. Ces formes pseudotumorales semblent survenir avec une plus grande fréquence au-delà de la cinquième décennie avec une légère prédominance féminine. Il s'agit donc d'une forme à déclaration tardive. Du point de vue pathogénique, ces lésions pseudo-tumorales sont attribuées à la confluence de granulomes venant comprimer les alvéoles de voisinage réalisant le syndrome pseudo-alvéolaire de Heitzman, il peut s'agir également d'une infiltration broncho-alvéolaire d'origine inflammatoire [8]. Sur le plan clinique, notre patiente se distingue par la discrétion de la symptomatologie fonctionnelle et la normalité de l'examen physique, contrastant avec le caractère multifocal et étendu des lésions observées. Cette constatation vient en accord avec celles de multiples séries où la grande majorité des patients étudiés étaient asymptomatiques au moment du diagnostic [1, 7]. Dans l'étude de Battesti portant sur 746 patients atteints de sarcoïdose, 21 patients parmi les 33 présentant une sarcoïdose pseudotumorale, soit 60% des sujets étaient asymptomatiques [9]. Dans la sarcoïdose pseudotumorale, la radiographie pulmonaire montre des opacités nodulaires de taille variable, souvent bilatérales réalisant parfois un véritable lâcher de ballon et associées à un élargissement médiastinal [1, 7]. Des atteintes pulmonaires unilatérales ont été également décrites. Il peut s'agir d'un nodule pulmonaire isolé, ou d'un aspect pseudo-tumoral hilaire. Le scanner thoracique montre des signes évocateurs ou compatibles lorsque la radiographie n'est pas évocatrice. On retrouve des images nodulaires, confluentes, infiltratives, bilatérales à limites mal définies, prédominant en périphérie, avec parfois un bronchogramme aérique traduisant leur nature alvéolaire [1], de taille variable (1 à 7 cm). Elles ne s'associent pas toujours à des adénopathies hilaires et/ou médiastinales [7].

La biologie n'est pas spécifique, un syndrome inflammatoire biologique modéré est fréquemment décrit. Les taux sériques de l'ECA ne sont pas toujours élevés. Ce qui ne diffère pas des autres formes de sarcoïdose. L'endoscopie bronchique est utile au diagnostic. Elle permet de visualiser d'éventuelles lésions macroscopiques nodulaires bronchiques et exceptionnellement des lésions sténosantes pseudotumorales [2]. Elle permet d'éliminer une origine néoplasique qui est souvent évoquée en premier lieu. Par ailleurs, la fibroscopie bronchique permet de confirmer la présence de lésions granulomateuses dans 57 à 88% des cas par biopsies de la muqueuse bronchique ou transbronchique [2]. Il n'existe aucune distinction fonctionnelle respiratoire entre ces formes pseudo-tumorales et les autres présentations de sarcoïdose [9]. L'évolution spontanée de ces formes pseudotumorales de la sarcoïdose demeure imprévisible et il n'existe à l'heure actuelle aucun critère prédictif certain quant à la réversibilité ou non des lésions installées. Dans certaines séries [1], l'évolution était spontanément favorable avec un délai de nettoyage radiologique allant de un à deux ans. Les formes nodulaires peuvent se compliquer d'excavation. Ces cavités peuvent elles-mêmes se compliquer de greffes aspergillaires exposant au risque d'hémoptysie [1, 6, 10]. Dans l'étude de Battesti [9], 22 des 33 patients présentant une sarcoïdose pseudotumorale ont été suivis. On note une disparition définitive des lésions chez 15 patients traités pendant 2 ans par corticoïdes. Chez six patients non traités, l'évolution était spontanément favorable avec un délai d'amélioration variable allant de trois à neuf mois. Un seul patient, pour lequel la corticothérapie était contre indiquée, avait développé une fibrose pulmonaire. Dans une série de 5 patients rapportés par Marques et al. aucun patient n'a été traité du fait du caractère asymptomatique des lésions radiologiques. L'évolution a été favorable dans tous les cas [1]. Il paraît donc au vu de ces données basées sur des séries souvent limitées, qu'il est encore difficile de préciser les indications et les modalités thérapeutiques [8]. Le traitement par corticoïdes ne semble pas apporter d'effet bénéfique dans les formes asymptomatiques [1] et ses indications restent celles des autres formes de sarcoïdose [6]. Les rechutes sont fréquentes à l'arrêt de la corticothérapie [7].

## Conclusion

La sarcoïdose peut exceptionnellement donner des images radiologiques en lâcher de ballon. La preuve histologique est nécessaire pour éliminer d'autres étiologies notamment tumorales. La discordance entre l'étendue des lésions radiologiques et la discrétion du tableau clinique doit attirer l'attention du clinicien. Du fait d'une évolution le plus souvent spontanément favorable, la prescription d'une corticothérapie ne paraît indiquée qu'en présence d'un retentissement fonctionnel respiratoire significatif et/ou d'une atteinte viscérale associée.
